# Using a Natural Clay Mineral as an Active Drug Carrier to Promote Hair Growth

**DOI:** 10.3390/ph19010011

**Published:** 2025-12-20

**Authors:** Zhiqing Liu, Wenhua Huang, Shanhua Xu, Meilan Nan, Xian Cui, Yue Wang, Zhehu Jin, Wan Meng, Jingbi Meng, Longquan Pi

**Affiliations:** 1 Department of Dermatology, Yanbian University Hospital, Yanji 133000, China; 2 Department of Medical Cosmetology, Yanbian University Hospital, Yanji 133000, China; 3 Department of Plastic Surgery, Yanbian University Hospital, Yanji 133000, China; 4 Department of Anesthesia, Yanbian University Hospital, Yanji 133000, China; 5 Department of Materials and Chemical Engineering, Yanbian University, Yanji 133000, China; 6 Yanbian University Research Center for Application of Illite Natural Clay (YURAI), Yanji 133000, China

**Keywords:** alopecia treatment, hair growth, illite, minoxidil

## Abstract

**Background:** Topical minoxidil remains the only FDA-approved treatment for hair loss, yet its clinical efficacy is compromised by organic-solvent-induced scalp irritation and poor patient adherence. This study aimed to evaluate natural illite as a carrier for minoxidil and to explore its potential hair-growth-promoting mechanisms. **Methods:** Thermal–acid-modified illite was engineered as a spray-dried, hydroalcohol-free minoxidil carrier for topical application. Hair regrowth efficacy was assessed in C57BL/6 mice via a 14-day depilation model. Mechanisms were elucidated via RNA-seq, Ki67/TUNEL immunofluorescence, and p-STAT3 immunohistochemistry. **Results:** Modified illite resulted in a 4.2-fold surface area increase and successful minoxidil loading. The minoxidil/illite formulation demonstrated efficacy equivalent to that of free minoxidil while also eliminating solvent toxicity. Mechanistic analysis revealed that illite functions as an active carrier: both the illite-alone and minoxidil/illite-treated groups exhibited increased Ki67^+^ proliferation and reduced TUNEL^+^ apoptosis. Transcriptomic profiling demonstrated dual mechanisms—enrichment of Myc proliferation pathways and suppression of IL-6 inflammatory signaling (*p* < 0.001)—with reduced p-STAT3 expression confirmed by immunohistochemistry. **Conclusions:** These findings suggest that an illite-based carrier can enable topical delivery of minoxidil with preserved efficacy and that illite itself exhibits intrinsic hair-growth-promoting activity via anti-inflammatory and pro-proliferative mechanisms, which may help alleviate adherence barriers associated with conventional topical alopecia therapy.

## 1. Introduction

Hair loss disorders constitute one of the most prevalent dermatological concerns globally, with significant psychosocial ramifications that extend across age, gender, and ethnic boundaries [1,2]. The clinical landscape encompasses a heterogeneous array of conditions—including androgenetic alopecia, alopecia areata, telogen effluvium, and chemotherapy-induced hair loss—each characterized by distinct pathophysiological mechanisms yet sharing a common therapeutic challenge: the need for safe, effective, and well-tolerated interventions [3,4].

Topical minoxidil remains the cornerstone pharmacotherapy for hair loss, distinguished by its broad-spectrum efficacy across multiple alopecia subtypes and unique status as the only FDA-approved topical agent for this indication [5]. Although initially developed as a systemic antihypertensive, minoxidil acts at the follicular level as a vasodilatory potassium channel opener and is converted locally to its active sulfate metabolite by follicular sulfotransferases [6]. Through these actions, minoxidil enhances perifollicular microcirculation, promotes dermal papilla cell survival, and prolongs the anagen phase, collectively supporting follicular regeneration and hair shaft production [6]. In routine clinical practice, minoxidil is formulated in hydroalcoholic solutions or foams that provide adequate solubilization and dermal delivery but frequently cause local burning, pruritus, and dryness due to the high content of ethanol and propylene glycol [7,8,9]. This efficacy–tolerability disconnect stems from reliance on organic-solvent-based formulations necessitated by minoxidil’s unfavorable physicochemical properties, creating a critical translational gap between pharmacological promise and real-world clinical outcomes. Accordingly, promising carriers for transdermal and follicular minoxidil delivery should combine suitable particle size, surface charge and specific surface area, as these physicochemical features critically influence drug loading, skin penetration, and residence within the pilosebaceous unit.

Topical minoxidil formulation faces fundamental physicochemical constraints: poor aqueous solubility necessitates high concentrations of organic cosolvents—primarily ethanol and propylene glycol—to achieve therapeutic dermal delivery [10,11]. While these vehicles effectively solubilize the drug, they simultaneously disrupt stratum corneum integrity, trigger inflammatory responses, and generate cosmetically unacceptable residues upon evaporation. The resultant cutaneous irritation—combined with twice-daily dosing requirements—undermines long-term adherence, creating a critical tolerability–efficacy disconnect that constrains clinical outcomes despite minoxidil’s robust pharmacological activity.

To address organic solvent-related limitations, alternative delivery strategies have been extensively explored, including lipid-based nanocarriers (liposomes, niosomes, solid lipid nanoparticles), polymeric platforms (biodegradable microspheres, hydrogels), and cyclodextrin inclusion complexes [12,13,14,15,16]. While these synthetic systems demonstrate promise in reducing cutaneous irritation and enabling controlled release, their clinical translation remains constrained by common translational barriers. Lipid-based formulations suffer from physicochemical instability, with phase separation and drug leakage during storage [16,17]. Polymeric carriers require complex manufacturing involving organic solvents, raising scalability and cost concerns [18]. Cyclodextrin complexes exhibit limited drug-loading capacity, necessitating impractically large excipient volumes [19]. These collective limitations underscore an unmet need for delivery platforms that integrate simplicity, biocompatibility, and scalability without compromising therapeutic performance—a gap that natural mineral carriers may uniquely address.

In addition to organic delivery platforms, inorganic materials such as clay minerals have been widely investigated as drug carriers. Clay–drug interactions have been exploited to modulate drug dissolution, stability, and release kinetics, and clay excipients are already used in marketed formulations [20]. In the hair and scalp field, halloysite clay nanotubes have been applied as biocompatible coatings that can deliver dyes or drugs to hair fibers, and recent reviews highlight inorganic nanomaterials alongside polymer- and lipid-based systems as promising carriers for cosmetic and therapeutic actives in hair-care and alopecia treatments [21,22]. These studies collectively show that clays are an established yet versatile class of drug delivery matrices.

Despite this progress, the use of natural clay minerals as carriers for clinically established hair-regrowth agents such as minoxidil, especially in hydroalcohol-free, dry-powder topical formats, remains relatively limited. Among phyllosilicate clays, illite—a non-swelling three-layer aluminosilicate abundant in sedimentary deposits—exhibits unique structural and physicochemical properties relevant to dermal drug delivery [23]. Its layered crystalline architecture allows for a large surface area and high cation exchange capacity, enabling efficient drug adsorption through electrostatic interactions [24]. Unlike montmorillonite and other swelling clays, illite maintains structural integrity across varying hydration states, offering formulation stability without phase transitions [25]. For instance, kaolin- and bentonite-based clays are routinely used as excipients in licensed dermal pastes, suspensions, and cleansing formulations, as well as in oral antidiarrheal products, highlighting their established safety in commercially available medicines. In this context, the potential of illite for lipophilic drug delivery stems from its amphiphilic surface chemistry. Isomorphic substitution within the aluminosilicate lattice generates permanent negative charges on basal surfaces, while edge sites expose hydroxyl groups with pH-dependent protonation, creating spatially heterogeneous interaction sites for both hydrophilic and lipophilic molecules [26]. This dual-affinity architecture has been exploited for the controlled release of various therapeutics, including anti-inflammatory agents and antimicrobials [27]. Critically, there are century-old safety records regarding clay minerals in pharmaceutical applications—from topical formulations to oral therapeutics—with established biocompatibility and a lack of systemic absorption following dermal application [20,28]. Moreover, reducing particle size to the submicron range enhances follicular penetration while preserving the mineral’s intrinsic properties, as demonstrated for other dermal applications [29,30]. Despite these compelling attributes, the application of illite-based carriers for minoxidil delivery—and indeed for hair restoration therapeutics broadly—remains unexplored, representing a significant knowledge gap at the intersection of advanced drug delivery and dermatological therapy.

To address this critical gap, we developed an illite-minoxidil composite particulate system and systematically evaluated its therapeutic efficacy. This natural mineral platform offers a biocompatible, hydroalcohol-free dry-powder alternative to ethanol-based formulations, providing a translational pathway toward improving adherence to minoxidil therapy.

## 2. Results

### 2.1. Enhancing the Surface Properties of Illite Through Thermal–Acid Modification

**Figure 1:**
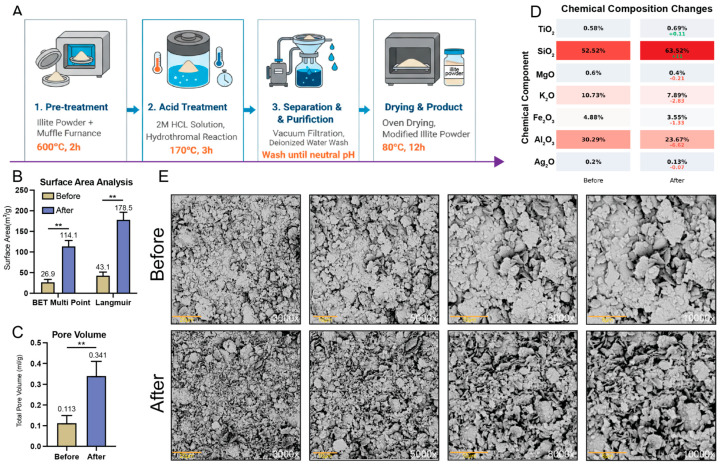
Illite’s structural and compositional changes after thermal–acid modification. (**A**) Schematic of the illite modification process: thermal calcination at 600 °C for 2 h followed by acid leaching in 2 M HCl at 170 °C for 3 h. (**B**) Specific surface area measured via N_2_ adsorption at 77k. The BET surface area increased from 26.9 ± 6.6 to 114.1 ± 13.8 m^2^/g, and the Langmuir surface area increased from 43.1 ± 8.2 to 178.5 ± 17.9 m^2^/g (n = 3). (**C**) Total pore volume determined at P/P_0_ = 0.99: 0.113 ± 0.036 mL/g (before) and 0.341 ± 0.069 mL/g (after) (n = 3). (**D**) Chemical composition (wt%) analyzed via XRF. Acid treatment selectively removed Al_2_O_3_ and K_2_O, enriching the SiO_2_ content. (**E**) SEM images showing morphological changes before (top) and after (bottom) modification. The original stacked lamellar structure became fragmented and irregular after treatment. Magnifications: 3000×, 5000×, 8000×, and 10,000×; Scale bars: 20, 10, 10, and 6 μm. ** *p* < 0.01.

Illite was modified via a two-step thermal–acid treatment, which enhanced its surface properties (Figure 1A). Nitrogen adsorption analysis showed a 4.2-fold increase in BET surface area (26.9 ± 6.6 to 114.1 ± 13.8 m^2^/g, *p* < 0.01) and a 4.1-fold increase in Langmuir surface area (43.1 ± 8.2 to 178.5 ± 17.9 m^2^/g; Figure 1B). Total pore volume increased 3-fold (0.113 ± 0.036 to 0.341 ± 0.069 mL/g, *p* < 0.01; Figure 1C). XRF analysis revealed removal of Al_2_O_3_ (30.3→23.7%) and K_2_O (10.7→7.9%), with concurrent SiO_2_ enrichment (52.5→63.5%; Figure 1D). SEM imaging showed transformation from densely stacked lamellar structures to fragmented, irregular platelets (Figure 1E).

### 2.2. Physicochemical Characterization of the Minoxidil/Illite Composite

**Figure 2:**
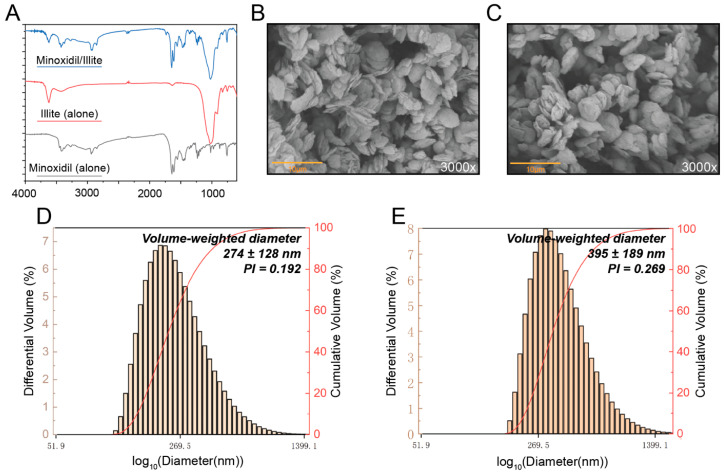
Physicochemical characterization of the minoxidil/illite composite. (**A**) FTIR spectra of illite (red), minoxidil (grey), and minoxidil/illite composite (blue). The composite retained the characteristic peaks of both components, confirming successful drug loading without structural degradation. (**B**,**C**) Representative SEM images of pristine illite (**B**) and the minoxidil/illite composite (**C**). (**D**) Particle size distribution of pristine illite measured via DLS. Volume-weighted diameter = 274 ± 128 nm; polydispersity index (PI) = 0.192. (**E**) The particle size distribution of the minoxidil/illite composite measured via DLS. Volume-weighted diameter = 395 ± 189 nm; PI = 0.269 (mean ± SD).

FTIR spectroscopy confirmed that minoxidil was successfully loaded onto illite. Pristine illite shows bands at ~1030 cm^−1^ (Si–O stretching) and 3624.8 cm^−1^ (Al–OH stretching), while the composite additionally displays minoxidil bands at 3420 cm^−1^ (N–H stretching) and 1650 cm^−1^ (C=N/N–H bending), indicating the coexistence of both components without disruption of the aluminosilicate framework (Figure 2A). The Al–OH band shifts from 3624.8 to 3618.6 cm^−1^ and decreases in intensity, and the OH/NH region becomes slightly broader, consistent with hydrogen bonding between illite Al–OH groups and amino groups of minoxidil at the clay–drug interface. SEM analysis suggested an overall lamellar morphology for both pristine illite and the minoxidil/illite composite. Within the resolution of our images, some regions of the composite appeared to exhibit slightly more separated layers and rougher, wrinkled surfaces compared with the predominantly stacked lamellae observed in pristine illite (Figure 2B,C). Although these morphological differences are modest, they are compatible with partial drug adsorption/intercalation at or between the layers and should be interpreted together with the FTIR and DLS data. Dynamic light scattering showed a 44% increase in particle diameter from 274 ± 128 nm to 395 ± 189 nm and elevated polydispersity from 0.192 to 0.269 (mean ± SD), consistent with drug adsorption and partial particle aggregation (Figure 2D,E). Zeta potential measurements showed that blank illite had a zeta potential of −30.6 ± 1.5 mV, whereas minoxidil-loaded illite exhibited −32.6 ± 0.8 mV (mean ± SD, n = 3). Both dispersions thus displayed |ζ| > 30 mV, which indicates relatively stability (Figure S1).

### 2.3. Quantitative and Histological Assessment of Hair Regrowth

**Figure 3:**
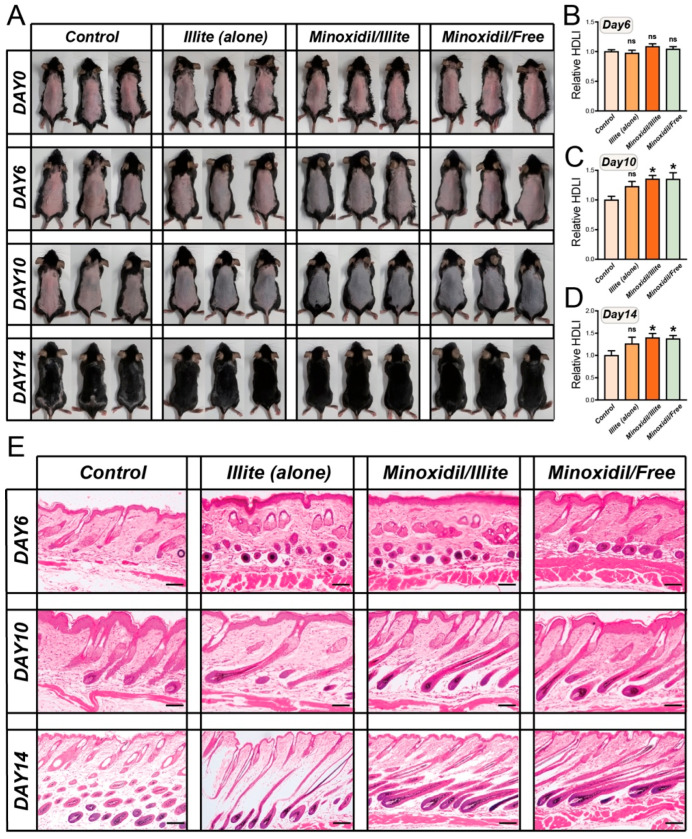
Temporal dynamics of hair regrowth and follicular cycling in response to treatments. (**A**) Representative dorsal photographs of the mice from each treatment group on days 6, 10, and 14 post-treatment. (**B**–**D**) Quantitative analysis of hair regrowth using relative HDLI on (**B**) day 6, (**C**) day 10, and (**D**) day 14. Statistical significance was determined via one-way ANOVA. * *p* < 0.05 vs. control. (**E**) Representative H&E-stained histological sections of dorsal skin on days 6, 10, and 14. Scale bars: 100 μm.

Hair regrowth was photographed on days 6, 10, and 14 (Figure 3A). No differences were observed on day 6 (Figure 3B). By day 10 (Figure 3C), both the Minoxidil/Illite and Minoxidil/Free groups demonstrated significantly higher HDLI levels than the control (*p* < 0.05). Notably, the Illite-only group showed a non-significant trend toward higher relative HDLI values compared with the control (*p* > 0.05). This trend may indicate potential hair-growth-promoting activity of Illite, but further studies with larger sample sizes are required to substantiate this hypothesis. This pattern persisted at day 14 (Figure 3D), with the Minoxidil/Illite treatment maintaining efficacy comparable to that of the Minoxidil/Free treatment, confirming that Illite does not compromise Minoxidil bioavailability. H&E staining revealed dynamic follicular cycling patterns (Figure 3E). On day 10, both Minoxidil groups displayed anagen VI follicles with deep hair bulbs and numerous epidermal openings, indicating synchronized anagen progression regardless of the delivery vehicle. Importantly, illite alone induced follicular elongation into the mid-anagen phases, demonstrating independent hair-growth-stimulating properties. By day 14, the control mice exhibited shallow telogen follicles, while both Minoxidil groups maintained anagen VI follicles upon entering early catagen with bulb retraction. Illite alone showed late catagen follicles, indicating transient but genuine anagen induction. These findings demonstrate that illite serves dual functions: it acts as an effective delivery vehicle that maintains Minoxidil’s bioactivity and as an active component with intrinsic hair-growth-promoting effects, albeit with shorter anagen duration than Minoxidil-based formulations.

### 2.4. Effects of the Treatments on Hair Follicle Cell Proliferation and Apoptosis

**Figure 4:**
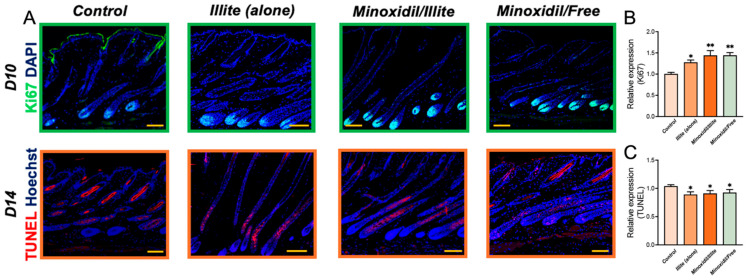
Proliferative and apoptotic dynamics in treated skin tissues. (**A**) Representative IF micrographs showing Ki67 (green) and TUNEL (red) signals with nuclear counterstaining (blue). (**B**) Relative expression of Ki67-positive cells on day 10 post-treatment. (**C**) Relative expression of TUNEL-positive cells on day 14. * *p* < 0.05, ** *p* < 0.01. Scale bar: 100 μm.

Representative confocal images of the Ki67 and TUNEL staining in murine dorsal skin are shown below (Figure 4A). Relative expression analysis revealed that on day 10, all the treatment groups exhibited significantly elevated populations of Ki67-positive proliferating cells relative to the controls (Figure 4B). Among these, the Minoxidil/Illite and Minoxidil/Free groups demonstrated the most pronounced proliferative enhancement (*p* < 0.01), whereas illite monotherapy induced a moderate but significant increase (*p* < 0.05). By day 14, TUNEL staining demonstrated a marked reduction in apoptotic cell populations across all treatment groups relative to the controls (*p* < 0.05; Figure 4C), indicating enhanced cellular survival. Because all mice were depilated on the same day during telogen and thus entered anagen synchronously, these differences at day 14 reflect treatment-associated modulation of apoptosis between time-matched groups rather than natural telogen progression alone.

### 2.5. Transcriptomic Profiling Reveals Distinct Molecular Responses to Treatments

**Figure 5:**
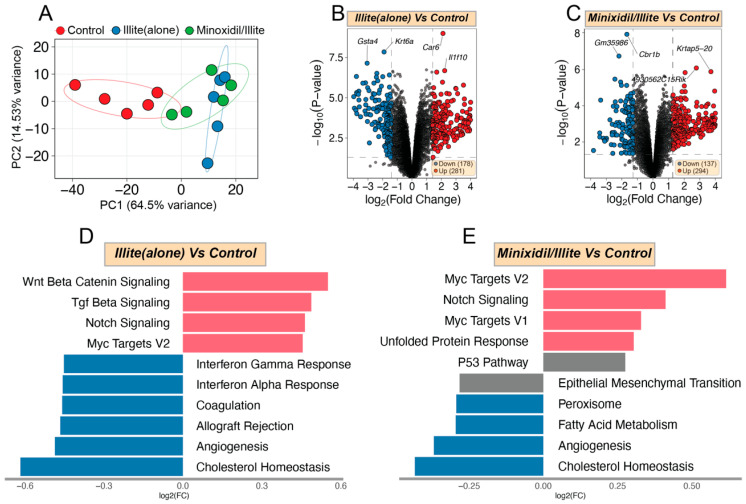
Transcriptomic analysis revealed Myc-driven proliferative signatures in illite and minoxidil/illite treatments. (**A**) PCA results showing distinct clustering of the control, illite, and minoxidil/illite treatment groups. (**B**,**C**) Volcano plots showing DEGs in the illite group versus the control (**B**) and the minoxidil/illite group versus the control (**C**) (black dots indicate non-significant genes). (**D**,**E**) GSVA bar plots depicting enrichment of Hallmark pathways in the illite (**D**) and minoxidil/illite (**E**) groups relative to the control. Both treatments show significant enrichment of Myc target gene signatures.

Principal component analysis (PCA) showed clear separation between the three groups, with PC1 and PC2 accounting for 79.03% of total variance (Figure 5A), indicating distinct transcriptional responses to different treatments. DEG analysis identified 459 significantly altered genes in the illite group relative to the control (281 upregulated and 178 downregulated (Figure 5B)) and 431 DEGs in the minoxidil/illite group relative to the control (294 upregulated and 137 downregulated (Figure 5C)). GSVA using Hallmark gene sets revealed significant enrichment of proliferation-related pathways in both treatment groups. Notably, the illite group showed strong activation of the Wnt/β-catenin, Notch, and Myc Targets V2 pathways (Figure 5D), while the minoxidil/illite group exhibited robust enrichment of the Notch, Myc Targets V1, and Myc Targets V2 pathways (Figure 5E). The prominent enrichment of Myc target gene signatures in both treatment groups is consistent with the significantly elevated Ki67 expression observed in the illite-treated and minoxidil/illite-treated tissues compared to the controls, suggesting that Myc-driven transcriptional programs may underlie the enhanced proliferative activity induced by these treatments.

### 2.6. Illite Suppresses IL-6-Mediated Inflammatory Signaling

**Figure 6:**
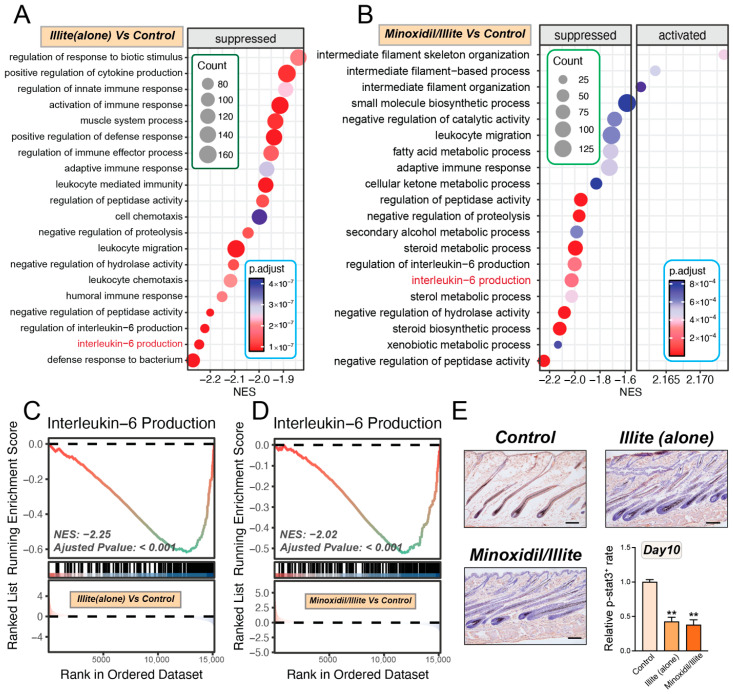
Illite suppresses IL-6-related pathways and p-STAT3 expression during hair regrowth. (**A**,**B**) GSEA of BP gene sets showing significant downregulation of multiple IL-6 production-related pathways in the (**A**) Illite (alone) vs. Control and (**B**) Minoxidil/Illite vs. Control groups. (**C**,**D**) Enrichment plots for the “Interleukin-6 production” pathway in the (**C**) Illite (alone) vs. Control (NES = −2.25, adjusted *p* < 0.001) and (**D**) Minoxidil/Illite vs. Control (NES = −2.02, adjusted *p* < 0.001) groups. (**E**) Representative IHC images and expression of p-STAT3 in dorsal skin sections on day 10 post-treatment. ** *p* < 0.01. Scale bar: 100 µm. NES, normalized enrichment score.

GSEA using the Biological Process (BP) dataset revealed that illite treatment alone significantly suppressed multiple IL-6-production-related pathways compared to the control (Figure 6A). Similarly, the combination of Minoxidil with Illite also induced pronounced inhibition of IL-6-associated inflammatory signaling cascades (Figure 6B). Focused analysis of the “Interleukin-6 production” gene set revealed robust negative enrichment in both treatment groups (Figure 6C,D). The Illite-Alone group showed a normalized enrichment score (NES) of −2.25 (adjusted *p* < 0.001), while the Minoxidil/Illite combination group exhibited an NES of −2.02 (adjusted *p* < 0.001), indicating significant downregulation of IL-6 production pathways in both intervention groups. We performed IHC staining for phosphorylated STAT3 (p-STAT3), a key downstream mediator of IL-6 signaling, in dorsal skin sections collected on day 10. Quantitative analysis revealed that both the Illite-Alone and Minoxidil/Illite combination treatments significantly reduced p-STAT3 expression levels compared to the control (*p* < 0.01) (Figure 6E), corroborating the RNA-seq results and confirming that Illite effectively attenuates IL-6/STAT3 inflammatory signaling in vivo.

## 3. Discussion

In this study, a minoxidil delivery system based on an illite carrier was developed, and, for the first time, its performance in promoting hair regeneration was demonstrated. Through animal experiments and molecular mechanism analyses, we found that this composite material achieved hair growth effects comparable to those of a hydroalcoholic minoxidil formulation in murine model, with indications of sustained biological activity.

Our HE staining results and the observed increase in the quantity of Ki67-positive cells, along with the decrease in the number of TUNEL-positive cells, are consistent with reports by Kim et al. (2022) and Oaku (2022) concerning minoxidil-induced hair growth promotion [31,32]. Similar proliferative responses have been documented with alternative delivery approaches, including liposomal minoxidil [33], polymeric microspheres [34], and cyclodextrin complexes [35], though with varying degrees of efficacy and duration [36,37]. These findings collectively corroborate the fundamental mechanisms of minoxidil’s action in stimulating hair follicle cell proliferation and reducing apoptosis, as extensively documented across multiple delivery platforms [36,37].

Our illite-based delivery system provides a novel formulation strategy for minoxidil while maintaining therapeutic efficacy comparable to that of a conventional hydroalcoholic formulation and showing favorable histological and molecular profiles in this model. These organic solvents, such as ethanol and propylene glycol in standard minoxidil products, have been reported in clinical studies to cause scalp irritation, contact dermatitis, and treatment discontinuation in a subset of patients. In contrast, in our animal experiments the Illite-based formulations did not show histological evidence of local toxicity and were associated with improved follicular proliferation and reduced apoptosis. Although direct head-to-head comparisons with advanced delivery platforms such as liposomes, hydrogels, or cyclodextrin complexes were beyond the scope of this proof-of-concept study, illite offers a simple mineral-based alternative that warrants further investigation.

While previous attempts to address solvent-related issues explored various approaches, these synthetic alternatives face significant translational challenges. Despite promising preclinical results, many of these systems suffer from scalability issues, with complex manufacturing processes that compromise cost-effectiveness and limited drug-loading capacity that necessitates frequent dosing [38,39]. More critically, the gap between animal model performance and human scalp physiology remains largely unaddressed, as most validation studies rely on murine models that fail to recapitulate the distinct characteristics of the human scalp, including a thicker stratum corneum and lower follicle density. Our illite-based system addresses these fundamental limitations by offering a naturally abundant, scalable carrier that demonstrates moderate drug loading without the manufacturing complexities inherently posed by synthetic alternatives. Most commercial minoxidil products rely heavily on organic solvents such as propylene glycol and ethanol to achieve adequate drug solubility and skin penetration [9]. While effective for drug delivery, these solvents are well-documented contributors to scalp irritation, contact dermatitis, and patient non-compliance, with reported incidence rates of 17.1% in clinical populations [7]. This solvent-related tolerability issue represents a significant barrier to long-term therapeutic success, often leading to treatment discontinuation despite clinical efficacy [40].

Beyond addressing tolerability concerns, our transcriptomic analysis at day 10 revealed that the illite–minoxidil system modulates inflammation-related pathways, including attenuation of IL-6/STAT3 signaling. These early anti-inflammatory changes may contribute to the enhanced efficacy and reduced irritation profile observed in this model. This stands in contrast to conventional hydroalcoholic formulations, in which organic solvents can actually exacerbate local inflammatory responses. The sustained biological effects observed at later time points raise the possibility of altered local pharmacodynamics of minoxidil when delivered via the illite-based carrier. However, we did not measure minoxidil concentration–time profiles or skin/follicular deposition; so, any impact on local drug residence time or dosing frequency remains speculative.

### Prospects and Challenges

Despite these promising findings, several limitations should be acknowledged. First, our study was conducted exclusively using animal models, and translation of these results to human subjects requires careful validation owing to potential species-specific differences in hair follicle biology and drug metabolism. Second, the relatively short observation period (2 weeks) does not allow for firm conclusions regarding the long-term safety profile and sustained efficacy of the illite-based system, particularly with respect to possible accumulation of mineral components within skin tissue and adnexal structures. Although our histological assessments did not reveal overt local toxicity, we did not perform elemental quantification of illite constituents in treated skin, and such analyses will be essential in future studies to rigorously characterize deposition and clearance kinetics. Third, our study focused on a single type of model, potentially limiting the generalizability of the findings to other forms of hair loss with different underlying pathophysiological mechanisms. Fourth, due to the scope of the present study, we did not include direct characterization of minoxidil release behavior or skin/follicular permeation from the illite formulation. As a result, any potential advantages in drug-delivery performance remain to be verified. Furthermore, although particle size distribution was quantified, drug loading efficiency and long-term formulation stability were not assessed. These aspects are important for fully defining the formulation’s behavior and will be addressed in follow-up studies.

Based on our mechanistic insights, there are several research directions that warrant investigation. The anti-inflammatory properties demonstrated by our transcriptomic analysis suggest potential applications beyond androgenetic alopecia, including inflammatory scalp conditions and chemotherapy-induced alopecia. The development of personalized clay-based delivery systems tailored to individual patient characteristics (scalp pH, sebum production, and inflammation status) represents an intriguing avenue for precision medicine in dermatology. Additionally, expanding this platform technology to other topical therapeutics could establish natural clay carriers as a versatile, biocompatible alternative to synthetic delivery systems. Most critically, well-designed clinical trials are needed to validate the enhanced efficacy and improved tolerability profile observed in our preclinical studies, potentially revolutionizing topical therapy for hair loss disorders.

These findings collectively suggest that our illite-based system may help address some of the formulation challenges that limit current minoxidil therapy and provides a mechanistically supported approach that warrants further evaluation for its potential to improve both efficacy and patient adherence in clinical practice.

## 4. Material and Methods

### 4.1. Materials Modification

Illite powder (Antu County, Yanji City, China) was calcined at 600 °C for 2 h (heating rate: 5 °C/min) in a muffle furnace. The calcined material (5 g) was transferred to a PTFE-lined stainless-steel autoclave containing 50 mL of 2 M HCl and heated at 170 °C for 3 h. After cooling, the suspension was vacuum-filtered and washed at least ten times with deionized water (pH 7.5–8.0). The conductivity of the filtrate approached that of the deionized water used for washing and showed no further decrease in three consecutive measurements. The solid was then dried at 80 °C for 12 h.

### 4.2. Surface Area and Porosity

N_2_ adsorption–desorption isotherms were measured at 77 K using a 3H-2000PM2 automated surface area and porosity analyzer (Beishide Instrument, Beijing, China). Samples (~150 mg) were degassed at 150 °C for 6 h under vacuum conditions before measurement. BET surface area was calculated from the linear region (P/P_0_ = 0.05–0.30), and total pore volume was determined at P/P_0_ = 0.99. Pore size distribution was obtained via BJH analysis of the desorption branch. The data represent the mean ± SD from n = 3 independent batches.

### 4.3. Elemental Analysis

Chemical composition was determined via X-ray fluorescence (XRF) spectroscopy using a Malvern Panalytical Epsilon 3 spectrometer (Malvern, UK). Samples were analyzed as pressed pellets under a helium atmosphere.

### 4.4. Fourier-Transform Infrared Spectroscopy (FTIR)

FTIR spectra were recorded on a Nicolet iS50 spectrometer (Thermo Fisher Scientific, Waltham, MA, USA) in the range of 4000–400 cm^−1^ with a resolution of 4 cm^−1^. Samples were prepared as KBr pellets by thoroughly mixing the sample with spectroscopic-grade KBr at a mass ratio of 1:150 (sample/KBr), followed by pressing the mixture into pellets under a pressure of 20 MPa. Each spectrum was obtained by averaging 32 scans.

### 4.5. Electron Microscopy

Scanning electron microscopy (SEM) was performed on a Phenom Pharos G2 field-emission scanning electron microscope operated at 5 kV. Samples were sputter-coated with an 8 nm Au/Pd layer prior to imaging.

### 4.6. Preparation of Minoxidil-Loaded Illite Composite Powder

Minoxidil-loaded illite composite powder was prepared via solvent evaporation followed by spray drying. Briefly, C8-10 mono- and diglycerides (>98%, Aladdin, Shanghai, China; 1.2 g) were dissolved in 30 mL of ethanol/propanol (3:1, *v*/*v*) under continuous stirring at 45 °C and 500 rpm for 10 min in amber bottles to prevent light degradation. Minoxidil (>98%, Aladdin, Shanghai, China; 1.3 g) was then added to the solution and stirred at 45 °C and 270 rpm for 3 h until complete dissolution. Illite powder (2.0 g) was subsequently added to the drug–surfactant solution and mixed at 50 °C and 270 rpm for 1 h. The mixture was homogenized at 10,000 rpm for 5 min using a high-speed disperser (Model T25, IKA, Staufen, Germany) and immediately spray-dried using a laboratory spray dryer (Model YC-1000, Shanghai Pilotech Co., Ltd., Shanghai, China) equipped with a 0.5 mm nozzle, at an atomization pressure of 0.05 MPa and a drying gas pressure of 0.2 MPa, with an inlet temperature of 120 °C, an outlet temperature of 80 °C, and a feed rate of 3 mL/min. The resulting powder was collected and stored in amber bottles until further use. Ethanol and propanol were used only as volatile processing solvents and were removed during spray drying, yielding a dry powder for topical application.

### 4.7. Preparation of Minoxidil-Loaded Illite Aqueous Dispersion

To prepare aqueous dispersions, we added the spray-dried composite powder (3.72 g) to distilled water (50 g) and stirred the combination overnight at 40 °C and 270 rpm to ensure complete hydration. The suspension was then homogenized at 12,000 rpm for 10 min to obtain a uniform 2.0 wt% minoxidil-loaded illite dispersion. The control formulations included unloaded illite dispersions (prepared with an equivalent amount of pristine illite powder) and ethanolic minoxidil solution (2% *w*/*v* in absolute ethanol) as a positive control. All dispersions were stored at 4 °C in amber bottles and used within 7 days to maintain stability.

### 4.8. Particle Size and Zeta Potential Measurement

The particle size, poly-dispersity index (PDI), and zeta potential of the micelles were evaluated using DelsaTMNano C Nanoparticle Analyzer (Beckman Coulter, Brea, CA, USA) in deionized water at 25 °C and pH 8.0.

### 4.9. Animal Models and Experimental Design

Female C57BL/6 mice (7 weeks old, ~20 g) were obtained from Liaoning Changsheng Biotechnology, were allowed to acclimatize for one week before the start of the experiment. They were housed under standard conditions with ad libitum access to food and water. All procedures were approved by the Animal Ethics Committee of Yanbian University (YD20250106009). Dorsal hair was removed via shaving followed by application of an animal depilatory cream (Animal Depilatory, Phygene, Fuzhou, China). The mice were randomized into four groups (n = 15/group): Control (vehicle; PBS), Illite-Alone, Minoxidil/Illite Composite, and Minoxidil/Free Solution. Topical treatments (500 μL per application) were administered once daily to the shaved dorsal area for 14 consecutive days. Among the 15 mice in each group, 3 animals whose macroscopic hair regrowth pattern during the early regrowth phase was closest to the group median were selected and used for imaging throughout the 14-day treatment period. At designated time points (days 6, 10, and 14), the mice were euthanized via isoflurane inhalation followed by cervical dislocation according to institutional guidelines. For RNA-seq analysis, on day 14 dorsal skin from 5 mice per group whose hair regrowth at that time point was closest to the group median was collected and processed for transcriptomic profiling.

### 4.10. Hair Growth Scoring and Image Analysis

Hair regrowth was quantitatively assessed using a modified Hair Density Logarithmic Index (HDLI) method with logarithmic transformation and control normalization. Digital photographs of the dorsal skin were captured on days 6, 10, and 14 post-treatments.

Image analysis was performed using an ImageJ macro. Images were first converted to 8-bit grayscale and subjected to automatic contrast normalization to reduce global differences in brightness and contrast across photographs. For each mouse, a standardized region of interest (ROI) was defined as a 2 × 2 cm square centered on the dorsal midline. When possible, an additional background reference ROI was selected on adjacent hairless skin within the same image, and a background-corrected logarithmic index HDLI_rel = log_2_(I_bg/I_hair) was calculated, where I_bg is the mean gray value of the background skin and I_hair is that of the hair-covered ROI. The HDLI algorithm incorporates two critical steps to enhance sensitivity and comparability:

Step 1—Logarithmic transformation: Raw HDLI values were calculated from grayscale intensity measurements within the ROI, reflecting both hair density and length. To linearize the relationship between perceived hair coverage and measured intensity, raw HDLI values were log-transformed:

Log−transformed HDLI=log2(Raw HDLI value)

Step 2—Control normalization: To eliminate inter-batch variability and facilitate cross-group comparisons, relative HDLI was computed by dividing each sample’s log-transformed HDLI by the mean log-transformed HDLI of the control group at the corresponding time point:

$$Relative\;HDLI\;=\frac{Log-\;transformed\;HDLI\;sample}{Mean\;Log-transformed\;HDLI\;control\;group}$$

The control group yielded a relative HDLI of 1.0 at each time point, serving as the normalization baseline. Values > 1.0 indicate enhanced hair growth compared to the control, while values < 1.0 indicate reduced growth. Three mice per group whose macroscopic hair regrowth pattern was closest to the group median were used for quantitative HDLI analysis. Statistical differences among groups were evaluated using one-way ANOVA as described in the Statistical analysis section.

### 4.11. Histological Analysis

Dorsal skin samples (4 mm punch) were collected on days 6, 10, and 14 from the treatment area; fixed in 4% paraformaldehyde for 24 h; and paraffin-embedded. The sections were deparaffinized, rehydrated, and stained with hematoxylin and eosin (H&E) according to standard protocols. The stained sections were examined under a light microscope equipped with a digital camera. Hair follicle morphology, follicular depth, and cycling stage were assessed according to established histological criteria [41]. Histological observations were qualitatively described and compared across groups.

### 4.12. Immunofluorescence (IF) Analysis of Proliferation and Apoptosis

The paraffin-embedded dorsal skin sections underwent deparaffinization followed by antigen retrieval. For Ki67 detection, sections were permeabilized using 0.3% Triton X-100 (15 min) and blocked with 10% goat serum (1 h). Rabbit monoclonal anti-Ki67 (1:200, C2305S, Beyotime, Shanghai, China) was applied overnight at 4 °C, followed by FITC-conjugated secondary antibody (1:500) for 2 h at ambient temperature. DAPI staining was employed for nuclear visualization. For apoptosis assessment, tissue sections were digested with Proteinase K (20 μg/mL, 37 °C, 30 min) prior to TUNEL labeling (C1088, Beyotime) at 37 °C for 60 min, with Hoechst 33342 serving as a nuclear counterstain. Fluorescence images were acquired using a Leica TCS SP5 II laser-scanning confocal microscope (Leica Microsystems GmbH, Wetzlar, Germany). Quantitative analysis of positive cells was conducted via ImageJ software (v1.54p).

### 4.13. Bulk RNA Sequencing and Analysis

Total RNA was extracted from the tissue samples (Day 10, n = 5 per group) using the TRIzol^®^ Reagent (Invitrogen, Waltham, MA, USA) according to the manufacturer’s protocol, with additional purification using an RNA Purification Kit (Cosmos Wisdom Co., Ltd., Hangzhou, China). RNA quality was assessed using a NanoDrop 2000 spectrophotometer (Thermo Fisher Scientific, Waltham, MA, USA) and an Agilent 2100 Bioanalyzer (Agilent, Santa Clara, CA, USA); only samples with RIN ≥ 7.0 and A260/A280 ratios of 1.8–2.0 were used. RNA-seq libraries were prepared using the Illumina Stranded mRNA Prep Ligation kit and sequenced by Cosmos Wisdom Co., Ltd. on an Illumina NovaSeq X plus platform. Raw reads were quality-controlled with FastQC (v0.12) and trimmed using Trimmomatic (v0.39). Clean reads were aligned to the human reference genome (GRCm39) using HISAT2 (v2.2.1), and gene-level counts were quantified with featureCounts (Subread v2.0.3) using Ensembl annotations. Differential expression analysis was performed using DESeq2 (v1.36.0) in R (v4.4.1). Genes with average counts <10 were filtered out, and principal component analysis was conducted on variance-stabilized transformed data. Differentially expressed genes (DEGs) were identified using the Wald test with Benjamini–Hochberg FDR correction (adjusted *p* < 0.05, |log_2_FC| > 0.5). Gene Set Variation Analysis (GSVA) was performed using the GSVA package (v1.44.0) with gene sets from MSigDB (v2024.1) [42,43]. We used the GSEA package in R to conduct Gene Set Enrichment Analysis (GSEA) against the Gene Ontology (GO) Biological Process (BP) dataset. Pathway enrichment significance was assessed using empirical Bayes-moderated *t*-tests (limma package) with Benjamini–Hochberg correction. Data were visualized using ggplot2.

### 4.14. Immunohistochemistry (IHC) and Quantification

Paraffin-embedded dorsal skin sections (day 10) were processed as described above. Following antigen retrieval in 10 mM sodium citrate buffer (pH 6.0, 20 min), endogenous peroxidase was quenched with 3% H_2_O_2_ (10 min). Sections were blocked with 5% normal goat serum (1 h) and incubated with anti-phospho-STAT3 (Tyr705) antibody (Abways, 1:200) overnight at 4 °C, followed by HRP-conjugated secondary antibody (1:500, 1 h). Signals were visualized using DAB substrate and counterstained with hematoxylin. Quantification was performed using ImageJ (n = 3 per group). One-way ANOVA was used for statistical analysis; *p* < 0.05 was considered significant.

## 5. Conclusions

In summary, we developed an illite-based minoxidil powder in which a natural clay mineral serves as an active drug carrier for topical hair-regeneration therapy. In a murine model, this composite achieved hair-regrowth efficacy that was similar to that observed with a hydroalcoholic minoxidil formulation. Mechanistic analyses, together with the moderate pro-regenerative effect observed for illite alone, suggest that the clay component is not merely an inert excipient but may exert intrinsic bioactivity, potentially involving proliferative and inflammatory pathways. While the present findings are limited to short-term treatment in mice and we did not perform quantitative residual solvent analysis or clinical irritation studies, our results support further investigation of clay-based composite carriers as complementary platforms for topical hair-regrowth therapies.

## Data Availability

The sequencing data generated in this study have been deposited in the National Genomics Data Center (NGDC) under project accession PRJCA038147. Further inquiries can be directed to the corresponding authors.

## Supplementary Materials

The following supporting information can be downloaded at: https://www.mdpi.com/article/10.3390/ph19010011/s1.

## References

[1] Christou E., Lalagianni N., McSweeney S.M., Cotter C., Ung C.Y., Walburn J., McCrone P., Turner M.A., McGrath J.A., Weinman J. (2025). Psychosocial burden and the impact of illness perceptions and stigma on quality of life, anxiety and depression in alopecia areata: Results from the Alopecia + Me study. Br. J. Dermatol..

[2] Jankowski G.S., Kranz D., Razum J. (2025). Men’s baldness stigma: A mixed methods international survey. J. Health Psychol..

[3] Messenger A.G., Rundegren J. (2004). Minoxidil: Mechanisms of action on hair growth. Br. J. Dermatol..

[4] Sattur S.S., Sattur I.S. (2021). Pharmacological Management of Pattern Hair Loss. Indian J. Plast. Surg..

[5] Devjani S., Ezemma O., Kelley K.J., Stratton E., Senna M. (2023). Androgenetic Alopecia: Therapy Update. Drugs.

[6] Gupta A.K., Talukder M., Venkataraman M., Bamimore M.A. (2022). Minoxidil: A comprehensive review. J. Dermatol. Treat..

[7] Kiratiwongwan R., Boonchai W., Kanokrungsee S. (2025). Allergic Contact Dermatitis to Topical Preparations Containing Minoxidil: A Systematic Review and Individual Participant Data Meta-Analysis. Dermatitis.

[8] BinJadeed H., Almudimeegh A.M., Alomran S.A., Alshathry A.H. (2021). A Case of Contact Allergic Dermatitis to Topical Minoxidil. Cureus.

[9] Rossi A., Cantisani C., Melis L., Iorio A., Scali E., Calvieri S. (2012). Minoxidil use in dermatology, side effects and recent patents. Recent Pat. Inflamm. Allergy Drug Discov..

[10] Pirhayati F.H., Mirzaeei S., Rahimpour E., Mohammadi G., Martinez F., Taghe S., Jouyban A. (2019). Experimental and computational approaches for measuring minoxidil solubility in propylene glycol + water mixtures at different temperatures. J. Mol. Liq..

[11] Tata S., Flynn G.L., Weiner N.D. (1995). Penetration of minoxidil from ethanol/propylene glycol solutions: Effect of application volume and occlusion. J. Pharm. Sci..

[12] Kochar P., Nayak K., Thakkar S., Polaka S., Khunt D., Misra M. (2020). Exploring the potential of minoxidil tretinoin liposomal based hydrogel for topical delivery in the treatment of androgenic alopecia. Cutan. Ocul. Toxicol..

[13] Souto E.B., Baldim I., Oliveira W.P., Rao R., Yadav N., Gama F.M., Mahant S. (2020). SLN and NLC for topical, dermal, and transdermal drug delivery. Expert Opin. Drug Deliv..

[14] Su Y., Zhang B., Sun R., Liu W., Zhu Q., Zhang X., Wang R., Chen C. (2021). PLGA-based biodegradable microspheres in drug delivery: Recent advances in research and application. Drug Deliv..

[15] Loftsson T., Brewster M.E. (2012). Cyclodextrins as functional excipients: Methods to enhance complexation efficiency. J. Pharm. Sci..

[16] Geszke-Moritz M., Moritz M. (2016). Solid lipid nanoparticles as attractive drug vehicles: Composition, properties and therapeutic strategies. Mater. Sci. Eng. C.

[17] Chutoprapat R., Kopongpanich P., Chan L.W. (2022). A Mini-Review on Solid Lipid Nanoparticles and Nanostructured Lipid Carriers: Topical Delivery of Phytochemicals for the Treatment of Acne Vulgaris. Molecules.

[18] Danhier F., Ansorena E., Silva J.M., Coco R., Le Breton A., Preat V. (2012). PLGA-based nanoparticles: An overview of biomedical applications. J. Control. Release.

[19] Real D.A., Bolanos K., Priotti J., Yutronic N., Kogan M.J., Sierpe R., Donoso-Gonzalez O. (2021). Cyclodextrin-Modified Nanomaterials for Drug Delivery: Classification and Advances in Controlled Release and Bioavailability. Pharmaceutics.

[20] Aguzzi C., Cerezo P., Viseras C., Caramella C. (2007). Use of clays as drug delivery systems: Possibilities and limitations. Appl. Clay Sci..

[21] Panchal A., Fakhrullina G., Fakhrullin R., Lvov Y. (2018). Self-assembly of clay nanotubes on hair surface for medical and cosmetic formulations. Nanoscale.

[22] Pereira-Silva M., Martins A.M., Sousa-Oliveira I., Ribeiro H.M., Veiga F., Marto J., Paiva-Santos A.C. (2022). Nanomaterials in hair care and treatment. Acta Biomater..

[23] Murray H.H. (2000). Traditional and new applications for kaolin, smectite, and palygorskite: A general overview. Appl. Clay Sci..

[24] Carretero M.I., Pozo M. (2009). Clay and non-clay minerals in the pharmaceutical industry: Part I. Excipients and medical applications. Appl. Clay Sci..

[25] Cuadros J. (2017). Clay minerals interaction with microorganisms: A review. Clay Miner..

[26] Tombácz E., Szekeres M. (2006). Surface charge heterogeneity of kaolinite in aqueous suspension in comparison with montmorillonite. Appl. Clay Sci..

[27] Ruiz-Hitzky E., Aranda P., Darder M., Rytwo G. (2010). Hybrid materials based on clays for environmental and biomedical applications. J. Mater. Chem..

[28] Viseras C., Aguzzi C., Cerezo P., Lopez-Galindo A. (2007). Uses of clay minerals in semisolid health care and therapeutic products. Appl. Clay Sci..

[29] Lademann J., Richter H., Teichmann A., Otberg N., Blume-Peytavi U., Luengo J., Weiss B., Schaefer U.F., Lehr C.-M., Wepf R. (2007). Nanoparticles–An efficient carrier for drug delivery into the hair follicles. Eur. J. Pharm. Biopharm..

[30] Ferreira-Nunes R., Cunha-Filho M., Gratieri T., Gelfuso G.M. (2021). Follicular-targeted delivery of spironolactone provided by polymeric nanoparticles. Colloids Surf. B Biointerfaces.

[31] Oaku Y., Abe A., Sasano Y., Sasaki F., Kubota C., Yamamoto N., Nagahama T., Nagai N. (2022). Minoxidil Nanoparticles Targeting Hair Follicles Enhance Hair Growth in C57BL/6 Mice. Pharmaceutics.

[32] Kim M.J., Seong K.Y., Kim D.S., Jeong J.S., Kim S.Y., Lee S., Yang S.Y., An B.S. (2022). Minoxidil-loaded hyaluronic acid dissolving microneedles to alleviate hair loss in an alopecia animal model. Acta Biomater..

[33] Alghamdi R., Alamoudi W., Daggag W., Bokhary A., Zelai N. (2024). Minoxidil nanoliposomes as a hair growth stimulator and a scalp disinfectant. Pak. J. Pharm. Sci..

[34] Yin M., Zeng Y., Liu H.Q., Zhang W., Wang C., Chen C., Li W. (2023). Dissolving Microneedle Patch Integrated with Microspheres for Long-Acting Hair Regrowth Therapy. ACS Appl. Mater. Interfaces.

[35] Liu Z., Li X., Xiong S., Xiao T., Jiao S., Chai G., Xu Y. (2025). Co-delivery of minoxidil and finasteride via ionic liquid and cyclodextrin-assisted in situ thermosensitive hydrogel for synergistic treatment of androgenic alopecia. Int. J. Pharm..

[36] Xu S., Zhou L., Zhao H., Li S. (2025). Advances in Transdermal Delivery Systems for Treating Androgenetic Alopecia. Pharmaceutics.

[37] Andrade J.F.M., Verbinnen A., Bakst A., Cunha-Filho M., Gelfuso G.M., Gratieri T. (2025). An update on nanocarriers for follicular-targeted drug delivery for androgenetic alopecia topical treatment. Expert Opin. Drug Deliv..

[38] Wan L., Wang M., Song Y., Sun G., Zhang R., Chen Z., Zhou Y., Ma K., Zheng R., Gluchman M. (2025). Multifunctional Hydrogel for the Treatment of Atopic Dermatitis: Current Advances and Translational Challenges. Eur. J. Pharm. Sci..

[39] Gupta M.K., Sansare V., Shrivastava B., Jadhav S., Gurav P. (2022). Comprehensive review on use of phospholipid based vesicles for phytoactive delivery. J. Liposome Res..

[40] Junge A., Radonjic-Hoesli S., Bossart S., Simon D., De Viragh P., Hunger R.E., Heidemeyer K., Seyed Jafari S.M. (2025). Contact Dermatitis Caused by Topical Minoxidil: Allergy or Just Irritation. Acta Derm. Venereol..

[41] Muller-Rover S., Handjiski B., van der Veen C., Eichmuller S., Foitzik K., McKay I.A., Stenn K.S., Paus R. (2001). A comprehensive guide for the accurate classification of murine hair follicles in distinct hair cycle stages. J. Investig. Dermatol..

[42] Hanzelmann S., Castelo R., Guinney J. (2013). GSVA: Gene set variation analysis for microarray and RNA-seq data. BMC Bioinform..

[43] Castanza A.S., Recla J.M., Eby D., Thorvaldsdottir H., Bult C.J., Mesirov J.P. (2023). Extending support for mouse data in the Molecular Signatures Database (MSigDB). Nat. Methods.

