# Supplementary figures and images for: Using a Natural Clay Mineral as an Active Drug Carrier to Promote Hair Growth
Source: Pharmaceuticals (Basel). 2025 Dec 20;19(1):11. doi: 10.3390/ph19010011 (PMC12845174; doi:10.3390/ph19010011)

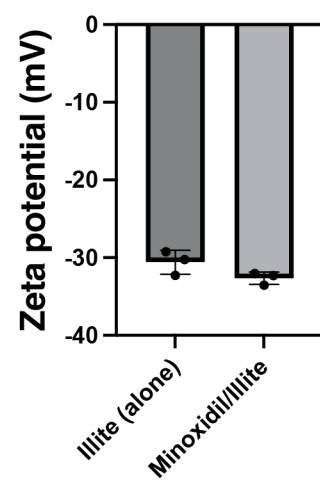

Figure S1 Zeta potential of blank illite and minoxidil-loaded illite

Supplement: Supplementary file 1 [file pharmaceuticals-19-00011-s001.zip › pharmaceuticals-4000469-supplementary.pdf]
